# Louisiana State Dental Society

**Published:** 1886-05

**Authors:** 


### LOUISIANA STATE DENTAL SOCIETY.

### ANNUAL MEETING FOR 1886.

REPORTED FOR THE INDEPENDENT PRACTITIONER BY "MRS. M. W. J."

(Concluded from page 205.)

Dr. W. J. Reese, an honorary member of the Society, read a long and valuable paper on

"URÆMIA, AND ITS EFFECTS UPON THE TEETH."

He was convinced, from the observations of a number of years, that uric acid in the blood was the cause of serious trouble in the mouth. Uric acid is found in the excrement of many reptiles, insects and birds of the carnivora, and of the herbivora while suckling. Dr. Beale has estimated the quantity of uric acid in healthy human urine to be from one-half to one grain in 1,000 grains, from five to eight grains being excreted daily by a healthy adult. Its presence is detected by the addition of a drop of nitric acid to the suspected deposit. If uric acid is present, brisk effervescence will take place. Evaporating over a lamp a reddish residuum is left, to which the addition of a drop of ammonia will give a rich purple tint, from the mur-oxide or purpurate of ammonia. Suspected fluid is to be treated with a few drops of glacial acetic acid, in a watch glass. Immersing filaments of silk or tow, and keeping them under a glass shade in a warm place, crystals of uric acid will be deposited on the filaments. Suspected saliva may be collected on a watch glass and evaporated over a spirit lamp, adding a drop of nitric acid and continuing the evaporation, stirring from the bottom with a stick dipped in ammonia. Uric acid, if present, will be thrown down as a red precipitate.

The proportion of uric acid in the urine varies with the diet used, being increased by a diet largely of meat, and also by the use of spirits. The percentage in various diseases has been estimated by Dr. Sansom to be in—

| | |
|---|---|
| Acute Gout | .830 |
| Rheumatism | .802 |

| | |
|---|---|
| Heart Disease | .711 |
| Erysipelas | .679 |
| Excessive Debility | .078 |
| In Health | .250 |

It is retained in the blood by diseased action of the kidneys, which lose their power of excreting uric acid, but eliminate it in health. During convalescence from gout or rheumatism large quantities are carried off in the urine and by the emunctories. "Fever blisters" are caused by the elimination of uric acid from the blood after fevers.

The homorrhagic diathesis is also intimately associated with uric acid troubles. Nasal catarrh, in connection with Pyorrhea Alveolaris, is probably due to the same cause. The effect of uric acid in the blood, upon the teeth, is a *phagedœna pericementi*—an eating away or absorption of the peridental membrane. Pyorrhea Alveolaris is a misnomer ; uric acid produces violent inflammation and intense pain, but rarely suppuration, except in contact with the fluids of the mouth, and not always even then. At the roots of the teeth, where protected from the air and the saliva not in contact, there is no suppuration. There is sometimes a bony deposit instead of absorption, and sometimes both of these conditions may exist about the same tooth, as in the superior molars, where, on the palatine root, the gum and alveolus will often be absorbed without suppuration, while the labial root will be exostosed, though apparently otherwise healthy, especially if it has no antagonist. The formation of tophus on the roots of the teeth is a concomitant of uric acid trouble, though not necessarily so. When present, absorption always precedes the deposit by from one-sixteenth to one-eighth of an inch. Sometimes the tophus of uric acid and deposits of salivary calculus—phosphate of lime—will be found on the same tooth, the latter being of a lighter color and more porous. Women, when irregular in menstruation, or during pregnancy and nursing, have, in addition to the deposits, the gum festoons purple, and bleeding at the slightest touch, sometimes from the quantity of blood and lack of inflammation, constituting a form of vicarious menstruation.*

*To check profuse hemorrhage after the extraction of teeth, take a very soft wax impression of the parts—trim it out and fill with a soft batter of plaster and apply immediately, pressing down firmly until the plaster is set. This will succeed after all other methods have failed.

The action of uric acid in dissolving the peridental membrane is the same as in the articulations and periosteum in other parts of the body, the peridental membrane being anatomically the same as the ligaments, as proved by the investigations of Dr. Acguilhen de Terran.

The systemic treatment is the same as for gout and rheumatism. Exercise in the open air, hot baths, keeping the skin clean and the pores open. Use vegetables and lemons freely, avoiding meat diet, as also alcoholic, vinous or malt liquors.

For local treatment, a surgical operation is rarely necessary. The urates dissolve in nitric or acetic acid; the efficacy of peroxide of hydrogen is due to the nitric and muriatic acid used in the manufacture. Uric acid yields its base to nitric acid, hence the peroxide of hydrogen dissolves deposits of recent formation, and by adding a small quantity of nitric acid the phosphate deposits are also dissolved, the nitric acid uniting with the alkaline base. Uric acid tophus can also be dissolved by potash, soda or ammonia, the alkalies uniting with the uric acid. It has also a great affinity for chloride of sodium, which will dissolve the deposits, but is very painful to the gums.

℞

| | |
|---|---|
| Creta Prep. | ℥ii |
| Bicarb. Soda (Squill's)<br>Pulv. South Amer. Soap—<br>Tree Bark | *aa* ℥i |
| Ol. Wintergreen | q. s. |

(Pulv. Orris Root may also be used).

This powder used freely by the patient, and packed around the gums at night, in connection with peroxide of hydrogen rubbed briskly over the deposits by the operator, will remove all deposits without the use of instruments.

The powder is also a specific for sensitive dentine at the gums. The pockets should be syringed with the peroxide, and the application made to the deposits by means of cotton wrapped on an excavator. The peroxide should not be exposed to the light, but the quantity necessary for one application should be poured into a clean vessel, from which the syring can be filled and into which the mop can be dipped. Robinson's Remedy is very efficacious, the potash neutralizing the uric acid, but it must be used with great caution.

℞

Hydrate Chloral
Spts. Cochlearea *aa* ℥ii applied with a mop, will be found equally efficacious, and safer if a napkin is used to protect the lips.

A paper was read by Dr. P. J. Friedrichs entitled

OBTURATORS.

He described the usual forms of Obturators and Artificial Velums—condemning the former as interfering with any efforts of nature towards closing the gap, while the latter, being soft and elastic, adapts itself to the varying surface of the palate, allowing the aperture to close by granulation, when properly protected.

He described a device of his own, by which the patient was able to exchange the flexible disk for a new one whenever it became softened, losing its elasticity. The patient was wearing a partial upper plate of vulcanite, to which he attached an elastic strip of 18 carat gold. In this strip he cut slots having two dove-tailed flanges, passing the strip through holes in the disk. The latter is held securely in place by the flanges, and easily removed when a new one becomes necessary. When a plate is not worn, the appliance can be attached to sound teeth by clasps.

Dr. J. W. Adams read a paper on

THE ORAL CAVITY AND ITS RELATIONS TO THE GENERAL SYSTEM.

He described in detail the mouth, its parts and their several functions, traced the intimate connection between the mouth and the nervous, circulatory and digestive systems, showing that diseases of the former were often the cause of obscure and persistent diseases, even of portions of the system apparently the most remote, the tongue affording an index to the condition of the stomach, the teeth of the condition of the uterus. Diseases of the blood and of the respiratory organs are caused by vitiated secretions from carious teeth, chronic abscesses or necrosed bone. He also cited cases of epilepsy, facial neuralgia, amaurosis, gout, hemorrhoids, diseases of the eye and ear, all resulting from diseases of the teeth and cured by proper attention to them.

Dr. J. G. McCulloch read a paper entitled

AMALGAM.

He said that, while in the hands of the charlatan and the quack it may be used to deceive and impose upon a confiding public, from the ease with which it is manipulated by those who have not the skill required to produce good results with gold, nevertheless, even the most skilled operators will find cavities so located that nothing else can be employed; teeth of a character that no other material will save, and patients whose means will not permit expensive operations in gold. He referred to an article published in the *Southern Dental Journal,* from the pen of G. Chisholm, in which various evil results and diseases are attributed to Amalgam fillings in the teeth, with, however, not a fact or a theory advanced to show how or why the Amalgam should be held responsible beyond the query: "If it was not the Amalgam, what was it?" He claimed that Amalgam stands second to no other material, when properly used, the cavity properly prepared and the filling thoroughly finished—used with judgment and in its proper place; that it would save teeth that no other material would save, and could be used where no other material could be employed. He spoke of its great value for attaching facial crowns to otherwise useless roots. Also as a means of saving the teeth of the poorer classes, who require assistance as much as more wealthy patients. If only for them, Amalgam is the greatest boon given to dentistry.

*Dr. J. S. Knapp*—Thought Amalgam should be used only when gold was out of the question; that it was not a durable material, being serviceable only for a few years; that being considered "cheap work" the fillings were not properly finished at the cervical margins and were not properly polished; also that Amalgam had a tendency to render the tooth-substance brittle, causing it to crumble away at the margins.

*Dr. O. Salomon*—Thought the brittleness, sometimes observed, due to copper in the Amalgam.

*Drs. G. J. and A. G. Friedrichs*—Thought all Amalgams had that effect.

*Dr. O. Salomon*—Uses pellets of old Amalgam, left over from the day before, impacted in soft, new fillings, to prevent shrinkage in very large fillings.

*Dr. J. R. Knapp*—Thought Amalgam objectionable, principally because of its uncertain character. It can not be depended upon. Sometimes it will bulge, sometimes it will shrink, and both results may be obtained from the same paekage and in the same mouth. Uses it with the matrix for partly filling very large cavities extending far under the gum, where it is next to impossible to so adjust the rubber dam as to permit the use of gold. In such cases the visible portions can be finished with gold, burring out the Amalgam after it is thoroughly hardened. In Amalgam fillings, dressing the margins and polishing the fillings are the most essential parts of the operation, and should be as thoroughly done as in the most expensive gold work, in both cases not for display, but for service and wear, for the preservation of the tooth and the comfort of the patient.

*Dr. A. G. Gayle*—Thought the combination of gold and Amalgam liable to create an electric current.

*Dr. Salomon*—Said that it had been demonstrated that where they were combined in one tooth, no such current was created.

*Dr. D. G. Parker*—Thought gold or even tin cylinders preferable to Amalgam. That Amalgam would bulge or move, and more or less would be left between the teeth, below the margins.

*Dr. J. R. Knapp*—Considered tin an excellent article, but requiring fully as much, if not more, skill and labor and space to handle than gold, in the inaccessible cavities to which he referred.

*Dr. A. G. Friedrichs*—Would use Amalgam only when it was impossible to use gold, and knew of no such limit except the inability of the patient to pay for gold. Would use gold exclusively, and as a rule in the form of cylinders. Did not use the rubber dam. Had a yard of it which had been in his office over a year. Had found no use for it.

Dr. G. J. Friedrichs, the President, calling the Vice-President to the chair, read a paper entitled

AQUA CALCIS.

This was a criticism of a paper published in The Independent Practitioner of November, 1885, entitled "Dental Nutrition." He quoted from that paper.

The election of officers resulted in the unanimous re-election of the President, Dr. G. J. Friedrichs, the Recording Secretary, Dr. Chas. Eckhardt and the Treasurer, Dr. C. C. Baquie. Dr. D. I. Parker was elected 1st Vice-President and Dr. M. J. Massingill, 2d Vice-President, and Dr. A. C. Gayle, Corresponding Secretary.

Adjourned to meet in New Orleans on the Wednesday after Mardi Gras, 1887.

